# Impact of renal impairment on outcomes after autologous stem cell transplantation in multiple myeloma: a multi-center, retrospective cohort study

**DOI:** 10.1186/s12885-018-4926-0

**Published:** 2018-10-20

**Authors:** Marlies Antlanger, Tobias Dust, Thomas Reiter, Alexandra Böhm, Wolfgang W. Lamm, Max Gornicec, Ella Willenbacher, David Nachbaur, Roman Weger, Werner Rabitsch, Susanne Rasoul-Rockenschaub, Nina Worel, Daniel Lechner, Hildegard Greinix, Felix Keil, Heinz Gisslinger, Hermine Agis, Maria-Theresa Krauth

**Affiliations:** 1 Department of Internal Medicine III, Division of Nephrology and Dialysis, Medical University of Vienna, Vienna, Austria; 2 Hanusch Hospital, 3rd Medical Department, Division of Hematology and Oncology, Vienna, Austria; 3 Elisabethinen Hospital, Department of Internal Medicine I, Division of Hematology and Oncology, Linz, Austria; 4 Department of Internal Medicine I, Division of Oncology, Medical University of Vienna, Vienna, Austria; 5 Department of Internal Medicine, Division of Hematology, Medical University of Graz, Graz, Austria; 6 Medical University of Innsbruck, Internal Medicine V, Hematology and Oncology, Innsbruck, Austria; 7 Department of Internal Medicine I, Bone Marrow Transplantation Unit, Medical University of Vienna, Vienna, Austria; 8 Medical University of Vienna, Center for Medical Statistics, Informatics and Intelligent Systems (CeMSIIS), Vienna, Austria; 9 Department of Blood Group Serology and Transfusion Medicine, Medical University of Vienna, Vienna, Austria; 10 Department of Internal Medicine I, Division of Hematology and Hemostaseology, Medical University of Vienna, Währinger Gürtel 18-20, 1090 Vienna, Austria

**Keywords:** Multiple myeloma, Renal impairment, Autologous stem cell transplantation, Overall survival, Progression-free survival

## Abstract

**Background:**

Renal impairment (RI) is a negative prognostic factor in Multiple Myeloma (MM) and affected patients are often excluded from autologous stem cell transplantation (ASCT). However, it remains unclear whether historically inferior outcome data still hold true.

**Methods:**

From a total of 475 eligible MM patients who had undergone ASCT between 1998 and 2016, 374 were included in this multi-centric retrospective cohort study. Renal function was determined both at the time of MM diagnosis and ASCT by estimated glomerular filtration rate (eGFR according to the MDRD formula, RI defined as eGFR < 60 ml/min/1.73m^2^). Patients were categorized into 3 groups: A) no RI diagnosis and ASCT, B) RI at diagnosis with normalization before ASCT and C) RI both at the time of diagnosis and ASCT. Log-rank testing was used for overall and progression-free survival (OS, PFS) analysis.

**Conclusion:**

While severe RI at MM diagnosis confers a risk of shorter OS, MM progression after ASCT is not affected by any stage of renal failure. It can be concluded that ASCT can be safely carried out in MM patients with mild to moderate RI and should be pro-actively considered in those with severe RI.

**Results:**

When comparing all groups, no difference in OS and PFS was found (*p* = 0.319 and *p* = 0.904). After further stratification according to the degree of RI at the time of diagnosis, an OS disadvantage was detected for patients with an eGFR < 45 ml/min/m^2^. PFS was not affected by any RI stage.

**Electronic supplementary material:**

The online version of this article (10.1186/s12885-018-4926-0) contains supplementary material, which is available to authorized users.

## Background

Multiple Myeloma (MM) is frequently accompanied and complicated by renal impairment (RI) [1, 2]. RI often develops secondary to cast nephropathy where urinary casts consisting of immunoglobulin light chains accumulate in the renal tubules [3]. Other potential causes include monoclonal immunoglobulin deposition disease, interstitial nephritis, tubular necrosis and proximal tubular damage resulting in secondary Fanconi syndrome [4]. Dehydration, hypercalcemia and administration of nephrotoxic medication often add to the development of acute RI [5–7]. Furthermore, as many patients are of advanced age at MM diagnosis, other chronic conditions such as arterial hypertension or diabetes mellitus can also underlie a chronic form of RI.

It is known that RI is associated with a higher rate of treatment-related toxicity and reduced overall survival (OS) [8, 9]. Outcomes are even worse when renal failure is advanced and dialysis support is required [10]. Regarding the clinical management, it is of pivotal importance to overcome the negative impact of MM-associated acute RI with prompt institution of anti-myeloma therapy and supportive measures such as adequate hydration, and treatment of metabolic acidosis [11–13].

Historically, RI has been defined in MM patients by a serum creatinine value above 2 mg/dL. In line with this classification, approximately 20% of all newly diagnosed MM patients were found to be affected [5]. Yet, as the normal creatinine range varies widely depending on a patients’ age, gender and muscle mass, this imprecise definition made the correct diagnosis and grading of RI difficult. As a result, the classification guidelines for RI in MM were adapted in 2014 [14]. The new criteria include renal function assessment by creatinine clearance measurement. Yet, this measurement also carries pitfalls and is less accurate than other formulas [15]. Alternatively, the estimation of glomerular filtration rate (eGFR) by the widely applied modification of diet in renal disease (MDRD) formula might represent an appropriate classification tool for renal function with a single measurement and is currently recommended by nephrologic guidelines for renal function assessment [16].

Since a higher frailty and transplant-related mortality have historically been postulated in MM patients with RI [17], they still often fail to qualify for high-dose induction chemotherapy and are excluded from autologous stem cell transplantation (ASCT). It seems noteworthy that analyses on which these approaches are based on were carried out applying old classifications of RI and were conducted before the era of immunomodulatory drugs (IMiD) and proteasome inhibitor-based therapy regimens [18]. Newer analyses have concluded that ASCT is safe in MM patients with RI [19].

Since the exclusion from or delay of ASCT results in shorter survival of MM patients per se [20, 21], it seems to be of pivotal clinical importance that MM patients with RI undergo early pro-active evaluation for high-dose immuno-chemotherapy and ASCT. To appraise the question of renal recovery rate following MM diagnosis and evaluate whether patients with initial RI benefit from ASCT, we analyzed the outcome of a multi-center cohort of MM patients with or without RI at diagnosis.

## Methods

### Patient recruitment

The present analysis was carried out as a multi-centric retrospective cohort study. Patients were eligible for inclusion if they had a diagnosis of MM according to the criteria of the International Myeloma Working Group and received a first ASCT between 1998 and 2016. Patients with relapsed disease receiving a second or third ASCT were excluded.

Data of 475 patients from five Austrian Bone Marrow Transplant units (Medical University of Vienna, Medical University of Innsbruck, Medical University of Graz, Hanusch Hospital Vienna, Elisabethinen Hospital Linz) were available for analysis. All centers participate in the Austrian Myeloma Registry (ethics committee number Innsbruck: AN 3252 266/4.2370/5.6 (3997a); further, the ethics committee of the Medical University of Vienna additionally #1085/2017 approved the analysis.

In cases where renal function parameters were unavailable either at the time of diagnosis or ASCT, the respective patient was excluded from the analysis (*n* = 101). The final analysis was performed on 374 patients.

### Renal function assessment

Renal function was assessed by serum creatinine measurement and a subsequent estimation of the GFR by the MDRD formula: eGFR = 175 × standardized serum creatinine^− 1.154^ × age^− 0.203^ × 1.212 [if black] × 0.742 [if female].

We then applied the KDIGO (Kidney Disease: Improving Global Outcomes) guidelines for renal failure and staged RI according to eGFR (cut-off for stage 2 at 90 ml/min/1.73m^2^, stage 3a at 60 ml/min/1.73m^2^, stage 3b at 45 ml/min/1.73m^2^ and stage 4 at 30 ml/min/1.73m^2^) [22].

Further, three subgroups were defined for analysis: A) always normal (eGFR > 60 ml/min/1.73m^2^ at diagnosis and ASCT), B) improving (eGFR < 60 ml/min/1.73m^2^ at diagnosis with normalization before ASCT) and C) always impaired (eGFR < 60 ml/min/1.73m^2^ both at the time of diagnosis and ASCT).

For supplementary analyses, a more stringent RI definition was chosen. Here, an eGFR value < 90 ml/min/1.73m^2^ defined a patient as being affected by RI.

### Induction therapy before ASCT

Prior to ASCT, all patients received induction (immuno)-chemotherapy. As therapy regimens were subject to change during the years of analysis and IMiDs as well as proteasome inhibitors became standard-of-care during the early 2000s, we categorized our patients into either receiving chemotherapy alone versus immuno-chemotherapy including an IMiD and/or proteasome inhibitor. No patients received monoclonal antibodies. Two-hundred ninety patients (77.5%) received the latter (containing either IMiD, proteasome inhibitor or both), while 84 patients (22.5%) received conventional chemotherapy (containing various combinations of cyclophosphamide, etoposide, doxorubicin, idarubicin, vincristine and bendamustin).

### Transplant procedure and transplant-related mortality

All ASCT procedures were carried out with peripheral blood stem cell grafts (2–4 × 10^6^ CD34+ cells/kg body weight). Conditioning regimens were melphalan-based in all patients: 84.7% received high-dose melphalan (200 mg/m^2^), 9.6% received a reduced dose (140 mg/m^2^), 4.7% received other doses of melphalan and 1.1% received melphalan and total body irradiation. Antimicrobial treatment as well as erythrocyte and platelet support were administered according to best clinical practice guidelines of the respective institution. OS was defined as survival from time of diagnosis, while PFS was defined as the survival free of disease progression or recurrence from the time of ASCT. As only 6 patients died of causes other than MM progression, the time to progression was not calculated separately, but described as PFS instead.

### Statistical analysis

We calculated cross-tables and Pearson chi-square tests for categorical variables and means and standard deviations as well as Analysis of Variance (ANOVA) models for continuous variables. Kaplan-Meier curves were generated for survival analyses and Log-rank tests were used to assess differences in OS and progression-free survival (PFS) between the study groups. A *p*-value ≤0.05 was considered statistically significant. The IBM SPSS System for Mac version 22.0.0 (SPSS, Inc., 2010, Chicago, IL) was used for all analyses.

## Results

### Renal function course between diagnosis and ASCT

The study cohort’s renal function parameters showed a distinct overall improvement between the time of diagnosis and ASCT, with the mean eGFR increasing from 68.8 ± 26.9 ml/min/1.73m^2^ to 81.7 ± 27.9 ml/min/1.73m^2^.

The largest group was constituted of 238 patients (64%) who always had an eGFR above 60 ml/min/1.73m^2^ (Group A, mean eGFR 83 ± 17 ml/min/1.73m^2^ at diagnosis and 93 ± 21 ml/min/1.73m^2^ at the time of ASCT). Group B consisted of 67 patients (18%) whose previously impaired renal function normalized during induction therapy (mean eGFR 42 ± 15 ml/min/1.73m^2^ at diagnosis and 82 ± 20 ml/min/1.73m^2^ at time of ASCT). Fifty patients (13%) always had an eGFR below 60 ml/min/1.73m^2^ (Group C, mean eGFR 33 ± 17 ml/min/1.73m^2^ to 41 ± 15 ml/min/1.73m^2^). Nineteen patients (5%) could not be categorized into any of the three pre-defined groups as they exhibited significantly inferior renal function at ASCT (eGFR 43 ± 23 ml/min/1.73m^2^) compared to diagnosis (eGFR 78 ± 16 ml/min/1.73m^2^, *p* < 0.001).

Overall, 13 patients (3%) qualified as having stage 5 renal disease at the time of diagnosis, while 29 (8%) had stage 4 and 75 (20%) were classified as stage 3.

### Patient characteristics at diagnosis

Group C patients were significantly older and had more advanced MM disease stages (Table 1). Patients presenting with a free light chain-only paraprotein were more likely to be categorized into Group C. β_2_ microglobulin was significantly higher in Groups B and C, while hemoglobin levels were lower in these groups (Table 2). Response rates to induction therapy were assessed after first-line therapy and proved comparable in all groups with an achievement of complete remission (CR), very good partial response (VGPR) or partial response (PR) in > 90% of all patients.

**Table 1:** Patient characteristics at diagnosis; primary hematological treatment

	Group A (*n* = 238)	Group B (*n* = 67)	Group C (*n* = 50)	*p*-Value
Characteristics				
Sex (% male)	58	54	56	0.368
Age (years)	54 ± 9	56 ± 9	59 ± 9	**0.003**
Monoclonal heavy chain (%)				
IgG	58	45	48	0.103
IgA	24	27	8	**0.031**
IgM	1	–	2	0.505
IgD	1	–	2	0.505
IgE	–	2	0	0.116
Free LC only	17	27	40	**0.001**
Kappa LC (%)	57	64	54	0.466
Osteolysis (%)	76	85	76	0.583
Clinical stage (ISS, %)				**< 0.001**
I	48	18	14	
II	33	24	30	
III	12	51	54	
BM infiltration (%)	46 ± 29	54 ± 29	55 ± 25	0.053
Therapy (%)				0.576
Immuno-chemotherapy	79	75	74	
Chemotherapy only	21	25	26	
Time between diagnosis and ASCT (months)	13 ± 18	10 ± 10	9 ± 7	0.145
Primary response (%)				0.607
CR	16	21	17	
VGPR	34	24	34	
PR	44	52	40	
SD	3	2	6	
PD	4	2	2	

*LC* light chain, *ISS* international staging system, *BM* bone marrow, *CR* complete remission, *VGPR* very good partial remission, *PR* partial remission, *SD* stable disease, *PD* progressive disease

*p* < 0.05: statistically significant

**Table 2:** Laboratory parameters at MM diagnosis

	Group A (*n* = 238)	Group B (*n* = 67)	Group C (*n* = 50)	*p*-Value
Characteristics				
β2 microglobulin (mg/L)	3.1 ± 1.8	6.9 ± 5.0	11.5 ± 15.1	**< 0.001**
Hemoglobin (g/dL)	11.9 ± 2.2	10.8 ± 2.1	10.7 ± 1.8	**< 0.001**
Calcium (mmol/L)	2.4 ± 0.3	2.6 ± 0.6	2.5 ± 0.5	**< 0.001**
Creatinine (mg/dL)	0.9 ± 0.2	1.9 ± 1.4	2.6 ± 1.8	**< 0.001**
eGFR (ml/min/1.73m^2^)	83 ± 17	42 ± 15	33 ± 17	**< 0.001**
Renal function stage (%)				**< 0.001**
1	30.7	–	–	
2	69.3	–	–	
3a	–	50.7	32.0	
3b	–	22.4	20.0	
4	–	23.9	26.0	
5		3.0	22.0	
Albumin (g/L)	39 ± 7	37 ± 8	40 ± 7	0.100
LDH (U/L)	175 ± 50	206 ± 105	201 ± 97	**0.002**
C-reactive protein (mg/dL)	1.1 ± 2.1	1.4 ± 2.6	2.0 ± 4.5	0.092

*eGFR* estimated glomerular filtration rate, *LDH* lactate dehydrogenase

*p* < 0.05: statistically significant

### Patient characteristics at ASCT

All patient groups showed significant improvement of renal function between MM diagnosis and ASCT (Table 3). β_2_ microglobulin remained higher in Group C, while it became comparable between Groups A and B. Similarly, hemoglobin levels became comparable in Groups A and B, while they remained lower in Group C. Patients who received a reduced dose of melphalan had lower eGFR rates at ASCT compared to those who received a standard dose (200 mg: 84.6 ± 26.1 compared to 140 mg: 61.6 ± 32.4 ml/min/1.73m^2^, *p* < 0.001). Hematological outcome after ASCT, which was assessed after 3 months, was comparable in all three groups.

**Table 3:** Laboratory parameters at the time of transplantation, ASCT-associated factors and hematological outcome

	Group A (*n* = 238)	Group B (*n* = 67)	Group C (*n* = 50)	*p*-Value
Characteristics				
Age (years)	55 ± 9	57 ± 10	59 ± 9	**0.018**
BM infiltration (%)	12 ± 16	19 ± 25	17 ± 21	0.097
β2 microglobulin (mg/L)	2.1 ± 0.7	2.5 ± 1.0	5.8 ± 6.9	**< 0.001**
Hemoglobin (g/dL)	12.1 ± 1.6	11.8 ± 1.9	10.8 ± 1.4	**< 0.001**
Calcium (mmol/L)	2.3 ± 0.2	2.2 ± 0.2	2.2 ± 0.2	0.191
Creatinine (mg/dL)	0.8 ± 0.2	0.9 ± 0.2	2.0 ± 1.6	**< 0.001**
eGFR (ml/min/1.73m^2^)	93 ± 21	82 ± 20	41 ± 15	**< 0.001**
Renal function stage (%)				**< 0.001**
1	50.4	20.9	–	
2	49.6	79.1	–	
3a	–	–	46.0	
3b	–	–	32.0	
4	–	–	12.0	
5			10.0	
Albumin (g/L)	39 ± 5	39 ± 5	39 ± 6	0.719
LDH (U/L)	178 ± 71	208 ± 131	180 ± 48	**0.033**
C-reactive protein (mg/dL)	0.4 ± 0.9	0.8 ± 2.0	0.9 ± 2.8	**0.035**
Days to 0.5 × 10^9^ ANC/L	11 ± 1	11 ± 1	11 ± 1	0.128
Days to 1.0 × 10^9^ ANC/L	12 ± 1	12 ± 2	12 ± 4	0.259
Days to 20 × 10^9^ platelets/L	11 ± 2	11 ± 3	12 ± 3	**0.001**
Days to 50 × 10^9^ platelets/L	16 ± 6	16 ± 6	19 ± 11	**0.008**
Dialysis during ASCT (%)	–	6	15	**< 0.001**
Response to ASCT (%)				0.300
CR	41	49	36	
VGPR	28	28	36	
PR	26	21	17	
SD	3	–	5	
PD	2	2	7	

*BM* bone marrow, *eGFR* estimated glomerular filtration rate, *LDH* lactate dehydrogenase, *ANC* absolute neutrophil count, *ASCT* autologous stem cell transplantation, *CR* complete remission, *VGPR* very good partial remission, *PR* partial remission, *SD* stable disease, *PD* progressive disease

*p* < 0.05: statistically significant

Thirteen patients required intermittent hemodialysis treatment during their hospital admission for ASCT including four patients from Group B and nine patients from Group C. These patients fared similarly with regard to OS and PFS compared to those who did not require dialysis.

### Transplant-related mortality

Three patients died within 100 days after ASCT. One female patient had early infectious complications from Pseudomonas aeruginosa requiring intensive care treatment and subsequently suffered from acute renal failure necessitating hemofiltration. Her eGFR at diagnosis had been 16 ml/min/1.73m^2^ and had improved to 75 ml/min/1.73m^2^ at ASCT. The second patient, who was from Group A, developed cholecystitis-related sepsis 3 months after ASCT and also required hemofiltration. However, he also had severe early extra-medullary progression of MM. In the third patient, who died 11 months after ASCT, no cause of death could be determined.

### Survival after ASCT according to renal function

**Fig. 1:**
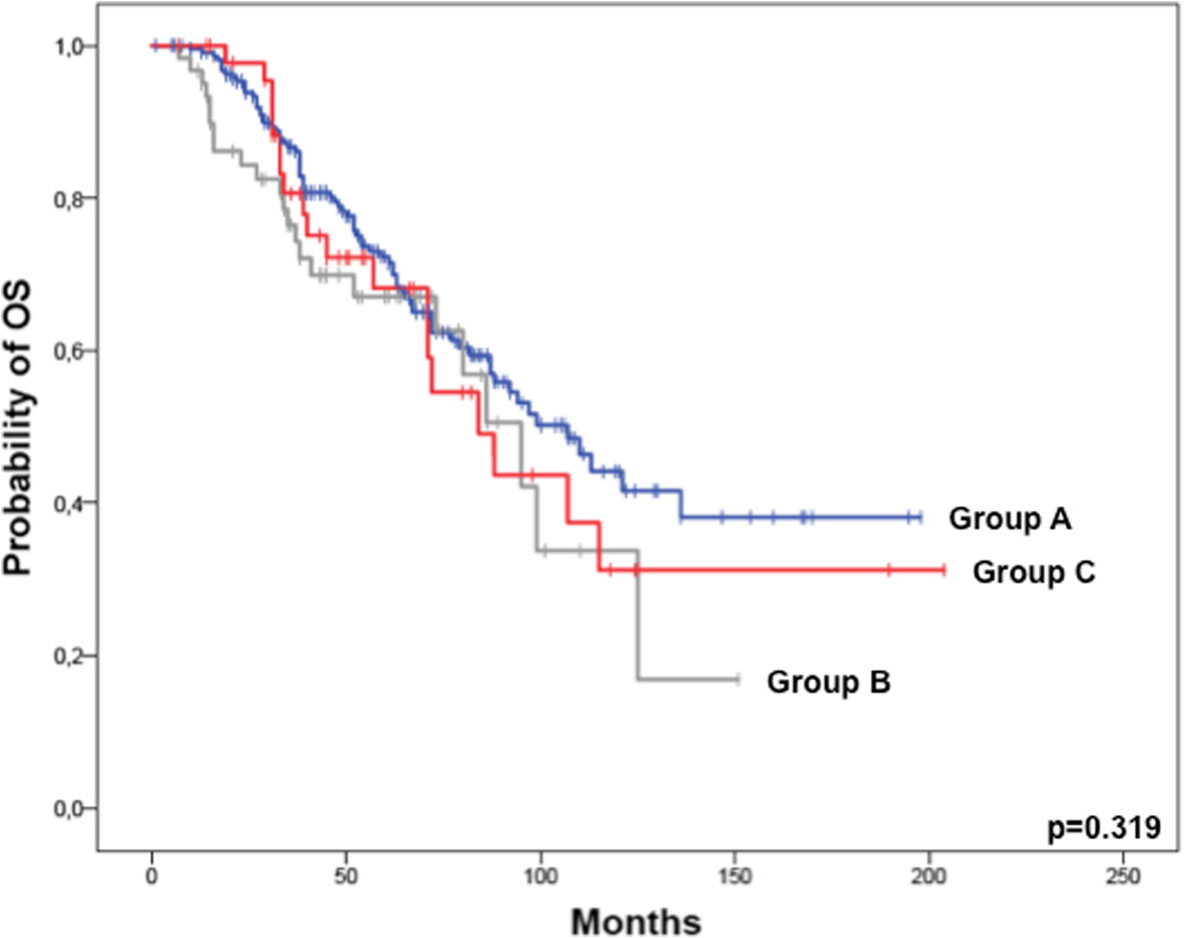
Overall survival (months) from the time of MM diagnosis according to renal function groups. Group A: eGFR always > 60 ml/min/1.73m^2^; Group B: eGFR < 60 ml/min/1.73m^2^ at diagnosis improving to > 60 ml/min/1.73m^2^ before ASCT; Group C: eGFR always < 60 ml/min/1.73m^2^

The 1-year OS rate was 94% in Group A, 97% in Group B and 98% in Group C (*p* = 0.348). It remained comparable after 3 years with rates of 70, 60 and 68%, respectively (*p* = 0.236). These differences did not amount to statistical significance on Kaplan-Meier survival analysis with Log-rank testing (Fig. 1**,** Log rank *p* = 0.319).

**Fig. 2:**
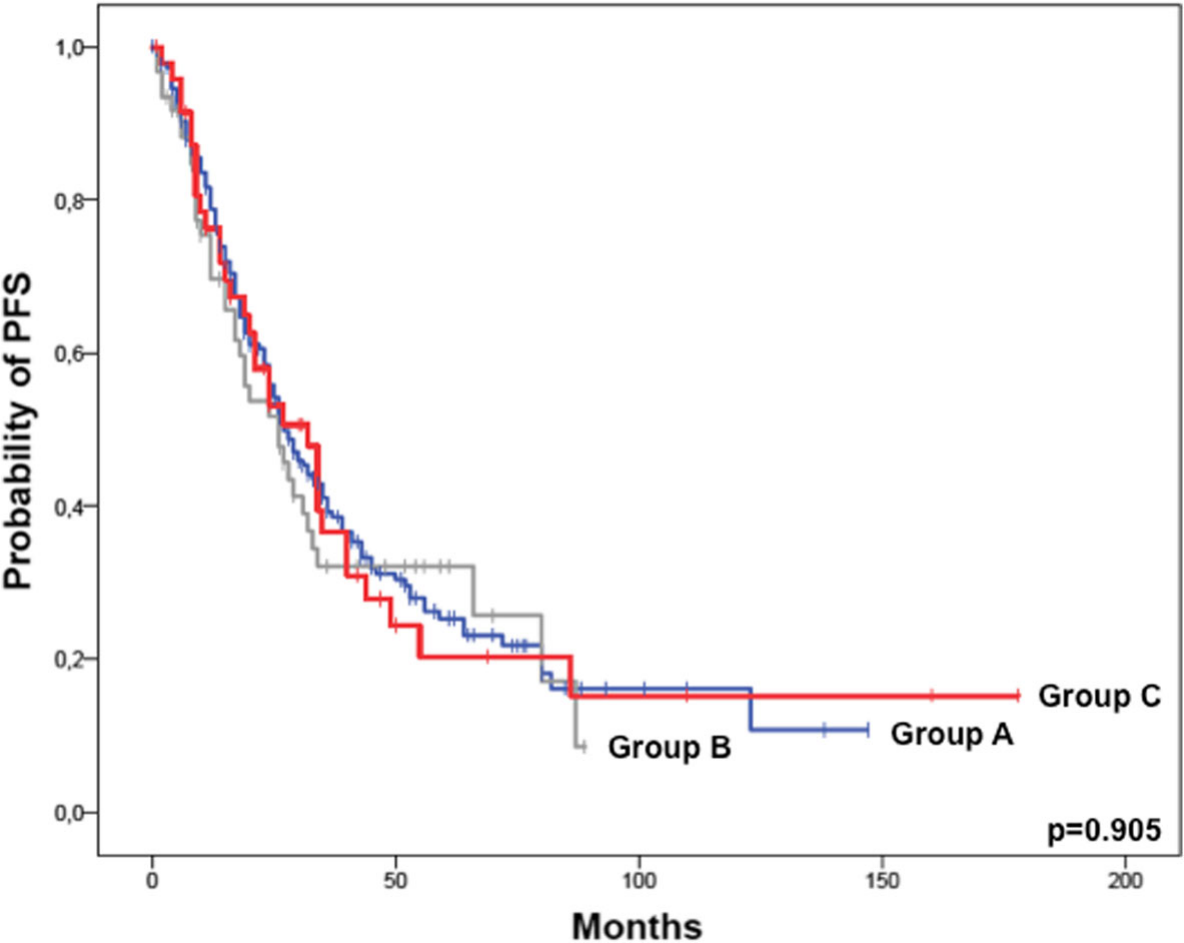
Progression-free survival (months) after ASCT according to renal function groups. Group A: eGFR always > 60 ml/min/1.73m^2^; Group B: eGFR < 60 ml/min/1.73m^2^ at diagnosis improving to > 60 ml/min/1.73m^2^ before ASCT; Group C: eGFR always < 60 ml/min/1.73m^2^

PFS rate at 1 year was 74% vs. 64% vs. 71% (*p* = 0.350), while the freedom of progression dropped to 29% vs. 23% vs. 27% at 3 years (*p* = 0.658). Again, no differences between the analyzed groups were observed on Log-rank testing (Fig. 2**,** Log rank *p* = 0.904).

**Fig. 3:**
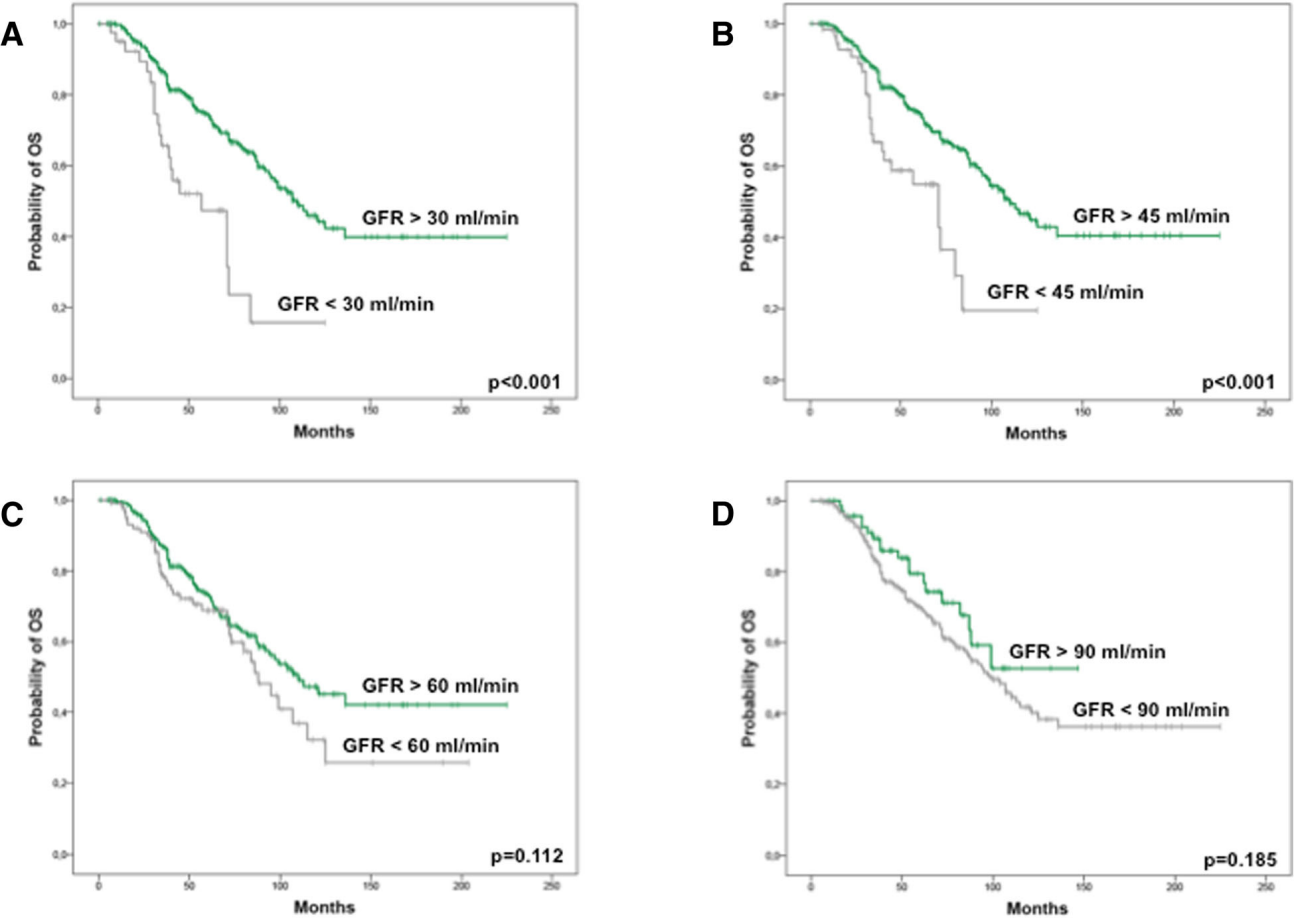
Overall survival (months) from the time of MM diagnosis according to renal function at diagnosis. **a** Stratification for eGFR above (green curve, *n* = 332) and below (grey curve, *n* = 42) 30 ml/min/1.73m^2^. **b** Stratification for eGFR above (green curve, *n* = 307) and below (grey curve, *n* = 64) 45 ml/min/1.73m^2^. **c** Stratification for eGFR above (green curve, *n* = 257) and below (grey curve, *n* = 117) 60 ml/min/1.73m^2^. **d** Stratification for eGFR above (green curve, *n* = 76) and below (grey curve, *n* = 298) 90 ml/min/1.73m^2^

**Fig. 4:**
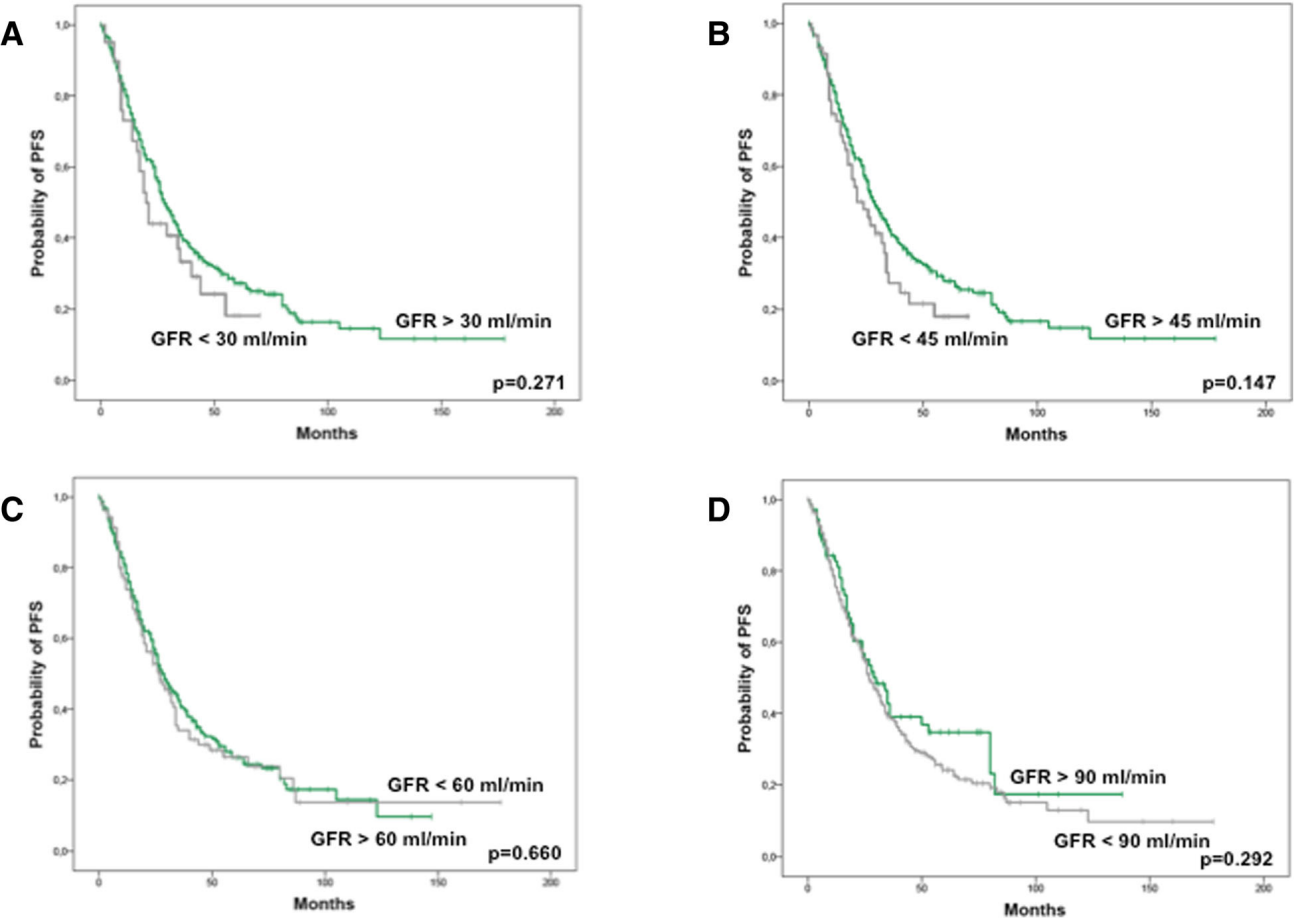
Progression-free survival (months) after ASCT according to renal function at ASCT. **a** Stratification for eGFR above (green curve, *n* = 332) and below (grey curve, *n* = 42) 30 ml/min/1.73m^2^. **b** Stratification for eGFR above (green curve, *n* = 307) and below (grey curve, *n* = 64) 45 ml/min/1.73m^2^. **c** Stratification for eGFR above (green curve, *n* = 257) and below (grey curve, *n* = 117) 60 ml/min/1.73m^2^. **d** Stratification for eGFR above (green curve, *n* = 76) and below (grey curve, *n* = 298) 90 ml/min/1.73m^2^

After further stratification according to RI stage at diagnosis, we found that eGFR < 30 ml/min/1.73m^2^ (corresponding to renal failure ≥ stage 4) as well as eGFR < 45 ml/min/1.73m^2^ (renal failure ≥ stage 3b) were significantly correlated with a shorter OS (Fig. 3a and b). Regarding PFS, no association between RI of any stage and survival free of hematological relapse was found (Fig. 4).

When including the small patient group who exhibited worsening of renal function during induction therapy, it was found that these patients did not exhibit different OS or PFS compared to the other groups (Log rank *p* = 0.066 and *p* = 0.721, data not shown).

Applying more stringent criteria for the definition of RI (eGFR below 90 ml/min/1.73m^2^), the group distribution shifted as expected: 14% of all patients were in Group A, 22% in Group B, 58% in Group C and 6% could not be classified. Yet, again, no significant differences between the groups could be determined on comparison of OS and PFS (Additional file 1: Figure S1).

On a further sub-analysis on OS and PFS comparing patients exhibiting an eGFR below 45 ml/min/1.73m^2^ at the time of ASCT with those above, no disadvantage was found for both outcomes (Log rank *p* = 0.629 and *p* = 0.927, data not shown).

## Discussion

Despite the high frequency of RI in MM and the extensive knowledge about its underlying pathophysiology, little is known about whether it poses a risk in the treatment with ASCT. The term ‘perceived frailty’, which has been coined by analyses describing hemodialysis patients [23], encompasses why many hematologists are hesitant when it comes to the evaluation for ASCT in patients with moderate to severe RI. Thus far, objective data on this issue remain scarce.

Here, we report on a multi-centric cohort of MM patients with varying degrees of underlying RI who received ASCT.

By definition of RI according to current guidelines, a substantial percentage of MM patients showed renal function impairment both at diagnosis and at the time of ASCT. Approximately one third of all patients had eGFR values below 60 ml/min/1.73m^2^ at diagnosis. Even though most of them had only mild to moderate RI with eGFR values above 30 ml/min/1.73m^2^, this finding substantially impacts clinical care of newly diagnosed MM patients. Similar findings have been described previously and correspond well to our results [24]. Early interdisciplinary care including an evaluation for kidney biopsy indication and the diligent treatment of electrolyte and acid-base disorders should be enforced in order to ensure the best treatment for these patients.

Second, a distinct improvement of renal function could be observed in many patients between the initial MM diagnosis and the time of ASCT. It cannot be concluded directly from the present data whether this development was achieved by the applied hematological induction therapy or by supportive care (e.g. discontinuation of pre-existent nephrotoxic medication, acid-base management during acute renal failure at the time of diagnosis, etc.); yet, a combination thereof must be suspected.

Third, the analyzed outcomes OS and PFS were highly comparable between patients whose renal function was always undisturbed, those who had RI at diagnosis but improved throughout the induction therapy phase and those whose renal function was always classified as impaired. Comparing these results to a previous analysis by San Miguel et al., a noticeable difference is the fact that they found an OS benefit in the group whose renal function – which at the time was defined by serum creatinine alone – had always been normal [25]. It can now be hypothesized that renal failure – both temporary and persistent – does not result in inferior hematological outcomes anymore as novel medications with fewer nephrotoxic effects, such as advanced immunotherapies, have become standard-of-care. This is in line with previous results by Scheid et al. who found treatment with bortezomib to result in an abrogation of the inferior PFS results in patients with impaired renal function [26]. In the context of significantly reduced OS in patients where ASCT is not considered at all or deemed too hazardous [20], it should be noted that patients with RI – even those with an eGFR below 45 ml/min/1.73m^2^ at the time of ASCT - clearly benefit from this treatment.

Fourth, the sub-groups of analyzed patients with moderately to severely impaired renal function at the time of diagnosis (defined as an eGFR below 45 ml/min/1.73m^2^ corresponding to renal failure stage 3b or worse) were found to have a decreased OS. Interestingly, this finding did not extend to hematological outcomes, as RI did not influence PFS. This confirms previously described results from Raab et al., who analyzed OS and PFS in a small cohort of 17 dialysis-dependent patients and compared them with a matched control group [27]. Similarly to our results, no difference in PFS was described. Although not statistically significant, OS was longer in dialysis-free patients in their analysis. Yet, this non-significance might be attributable to their very small sample size. Additionally, further data supporting the idea that severe RI is associated with shorter survival in MM patients has previously been delivered by analyses that defined RI by a serum creatinine value above 2 mg/dl, which can safely be interpreted as severe RI nowadays [28]. Considering our results and the well-known fact that renal failure is generally associated with a reduced life expectancy [29], an increased risk of earlier death in MM patients with severe RI should be acknowledged. However, our results cannot provide a new threshold definition for renal impairment in MM due to the limited sample size.

Some further limitations of this study warrant discussion: as the analysis was of retrospective nature and as only patients who actually received ASCT were included, patient selection bias cannot be ruled out. Further, the number of patients presenting with an eGFR < 60 ml/min/1.73m^2^ was small overall (*n* = 117), leading to limited power of the study. Additionally, induction therapy prior to ASCT was heterogeneous and we only analyzed effects of conventional chemotherapy versus immunochemotherapy including IMiDs and/or proteasome inhibitors. It must be suspected from previous studies that the choice of agent exerts a certain influence on renal function. Furthermore, as the graded measurement of spot urine albuminuria was only included into the KDIGO guidelines in 2009, we did not have enough proteinuria measurements at hand to provide substantial information on this aspect of renal impairment. Last, the definition and classification of RI in MM should remain a subject of critical discussion. Nowadays, nephrologic guidelines include eGFR measurements in their definition of chronic renal failure. The KDIGO grading of chronic kidney disease stages represents a simple and well-established tool; yet, other calculations besides the here-applied MDRD formula might be even more accurate in the estimation of renal function [30]. Furthermore, certain forms of renal failure, such as acute renal failure, are defined by different criteria (e.g. RIFLE, AKIN criteria [31, 32]), which makes a correct classification of MM patients, who can either be affected by acute or chronic renal disease, difficult. For our analysis, we consciously decided to use eGFR values and the grade of RI according to the KDIGO guidelines as a differentiation between acute and chronic RI was not fully possible in this cohort and, further, many patients actually did fulfill criteria for chronic renal failure (eGFR below 60 ml/min/m^2^ for ≥3 months).

## Conclusions

In conclusion, our data show that ASCT can be carried out safely in patients who present with mild to moderate renal failure at the time of diagnosis. Patients presenting with severe impairment of renal function should be pro-actively evaluated for ASCT, since hematological outcomes are comparable to those of patients with normal renal function. Further, while amelioration of renal function represents a highly desirable treatment goal, the lack of response should not preclude patients from autologous transplantation. Interdisciplinary care should be enforced in order to improve not only hematological, but also overall outcomes.

## Additional file

Additional file 1: **Figure S1.** Overall survival from MM diagnosis (**S1A**) and progression-free survival from ASCT (**S1B**) in months according to renal function groups. RI was defined as eGFR < 90 ml/min/1.73m^2^. Group A: eGFR always > 90 ml/min/1.73m^2^; Group B: eGFR < 90 ml/min/1.73m^2^ at diagnosis improving to > 90 ml/min/1.73m^2^ before ASCT; Group C: eGFR always < 90 ml/min/1.73m^2^. (TIF 1521 kb)

## Abbreviations

ASCT: Autologous stem cell transplantation
CR: Complete remission
eGFR: estimated glomerular filtration rate
IMiD: Immunomodulatory drug
KDIGO: Kidney disease improving global outcomes
MDRD: Modification of diet in renal disease
MM: Multiple Myeloma
OS: Overall survival
PFS: Progression-free survival
PR: Partial response
RI: Renal impairment
VGPR: Very good partial response
