# Supplementary figures and images for: Impact of renal impairment on outcomes after autologous stem cell transplantation in multiple myeloma: a multi-center, retrospective cohort study
Source: BMC Cancer. 2018 Oct 20;18:1008. doi: 10.1186/s12885-018-4926-0 (PMC6195957; doi:10.1186/s12885-018-4926-0)

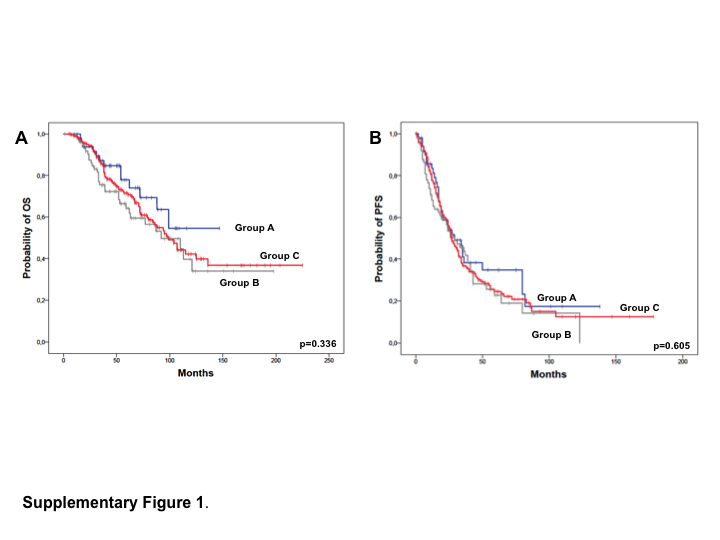

Supplement: Supplementary file 1 — Figure S1. Overall survival from MM diagnosis (S1A) and progression-free survival from ASCT (S1B) in months according to renal function groups. RI was defined as eGFR < 90 ml/min/1.73m2. Group A: eGFR always > 90 ml/min/1.73m2; Group B: eGFR < 90 ml/min/1.73m2 at diagnosis improving to > 90 ml/min/1.73m2 before ASCT; Group C: eGFR always < 90 ml/min/1.73m2. (TIF 1521 kb) [file 12885_2018_4926_MOESM1_ESM.tif]
